# Epidemiological intricacies of respiratory pathogens: a single-center study on infection dynamics in Beijing, 2023–2024

**DOI:** 10.3389/fpubh.2025.1581815

**Published:** 2025-06-26

**Authors:** Wang Jie, Huo Wenyan, Liu Chang, Feng Jinghong, Li Wenyi, Li Shanshan, Su Ming

**Affiliations:** ^1^Department of Clinical Laboratory, Peking University People's Hospital, Beijing, China; ^2^Department of Clinical Laboratory, Affiliated Hospital of Inner Mongolia Minzu University, Mongolia, China

**Keywords:** SARS-CoV-2, respiratory pathogens, co-detection, surveillance, diversity

## Abstract

**Background:**

Following China’s post-COVID-19 reopening strategy, intermittent small-scale outbreaks of respiratory pathogen infections have been observed in the Beijing region. This single-center study aims to characterize the epidemiological features and co-detection patterns of respiratory pathogens in Beijing, providing a scientific basis for the prevention and control of respiratory infectious diseases.

**Methods:**

We performed a retrospective analysis of 19,535 patients undergoing SARS-CoV-2 testing and 12,372 patients screened for six respiratory pathogens, including *Mycoplasma pneumoniae* (MP), respiratory syncytial virus (RSV), human rhinovirus (HRV), influenza A virus (IAV), adenovirus (ADV), and influenza B virus (IBV) at Peking University People’s Hospital from January 2023 to December 2024. Epidemiological data were systematically collected and analyzed.

**Results:**

SARS-CoV-2 positivity rate maintained above 3% throughout the observation period. IAV demonstrated distinct seasonal peaks in March 2023 (41.9%, 52/124), November–December 2023 (24.5%, 119/485 to 21.9%, 208/950), and December 2024 (25.4%, 289/1136). Poly-epidemics of multiple pathogens emerged between October 2023 and April 2024. Pediatric populations showed highest prevalence of MP (21.4%, 158/740), co-detection (15%, 111/740) and ADV (12.7%, 94/740), while adults predominantly exhibited SARS-CoV-2 (35.1%, 674/1920), RSV (15.8%, 304/1920) and IAV (12.3%, 236/1920). Among 324 co-detection cases, MP (130 cases), HRV (117 cases), and IAV (111 cases) were most frequently involved, with MP + HRV (36 cases), MP + IAV (31 cases), and HRV + IAV (23 cases) being the predominant pairwise combinations.

**Conclusion:**

Beijing experienced alternating waves of respiratory pathogen epidemics and co-detections during 2023–2024. IAV maintained characteristic winter–spring seasonality, while MP emerged as the predominant pathogen in co-detection events. Distinct pathogen profiles between pediatric and adult populations underscore the necessity for continuous surveillance and age-specific prevention strategies for respiratory infections.

## Introduction

The COVID-19 pandemic since 2019 has imposed unprecedented challenges on global public health and socioeconomic systems. In China, where rigorous Non-Pharmaceutical Interventions (NPIs) were maintained until December 2022, only sporadic or regional outbreaks of infectious diseases occurred during this period (1). These prolonged containment measures substantially suppressed the transmission dynamics of seasonal respiratory pathogens, including influenza and respiratory syncytial virus (RSV) (2–4). This epidemiological shift was particularly evident in Beijing – a megacity with 21 million residents in northern Chinese. The sustained suppression of pathogen circulation disrupted seasonal transmission patterns, creating an immunity gap that heightened population susceptibility to respiratory infections (2, 5).

Seasonal influenza remains a global health burden, causing approximately 500,000 annual deaths worldwide. Current epidemics predominantly involve antigenically evolving influenza A (IAV) and influenza B (IBV) viruses, whose continuous antigenic drift enables immune evasion and transmission complexity (6). RSV exhibits winter-seasonal predominance in temperate regions, primarily causing lower respiratory tract infections in children under 2 years (7). *Mycoplasma pneumoniae* (MP) mainly demonstrates spring-seasonal peaks (8). While human rhinovirus (HRV) shows bimodal spring-autumn activity, both HRV and adenovirus (ADV) maintain year-round circulation without distinct seasonality (8). Pathogen transmission dynamics are further modulated by regional climatic and sociocultural factors (2). Following China’s pandemic policy shift in December 2022, a surge of Omicron variants emerged (9), succeeded by intermittent clusters of febrile respiratory illnesses across Beijing throughout 2023. This resurgence coincided with intensified respiratory pathogen surveillance. Unlike nations that implemented earlier reopenings, China’s prolonged “dynamic zero-COVID” strategy (2020–2022) created unique epidemiological conditions. Numerous studies have emphasized the necessity of strategic vaccination and surveillance programs to address potential immunity debt and mitigate future infection surges (2, 10, 11). Consequently, characterizing post-pandemic respiratory pathogen epidemiology is crucial for developing targeted intervention strategies.

This retrospective study analyzed clinical specimens collected from Peking University People’s Hospital between January 2023 and December 2024, comprising two distinct cohorts: 19,535 cases undergoing SARS-CoV-2-specific testing and 12,372 cases screened for a six-pathogen panel, including IAV, IBV, MP, HRV, RSV, ADV. As part of routine testing in our hospital and clinically common respiratory pathogens (10), we systematically investigated the epidemiological evolution and transmission patterns of these seven common respiratory pathogens (including SARS-CoV-2) in the post-pandemic era by employing nucleic acid detection methods. The study objectives were threefold: (1) to delineate temporal trends in pathogen prevalence, (2) to identify co-circulation patterns among respiratory pathogens, and (3) to establish an evidence base for optimizing public health strategies. Our findings will provide critical insights for enhancing surveillance systems, refining vaccination protocols, and developing targeted prevention measures.

## Materials and methods

### Study population and data collection

This study was conducted in Peking University People’s Hospital from January 1st, 2023 to December 31th, 2024. This hospital is located in Beijing, the capital of China, which is a typical temperate monsoon climate region featuring with distinct four seasons and relatively dry climate. We retrieved the test results of IAV, IBV, MP, HRV, RSV, ADV and SARS-CoV-2 during this period, and combined with the corresponding patient ID, which symbolizes unique identifiers of different individuals, and other information such as sex, age and sample collecting data. Doctors mainly determined these test items according to the respiratory infection symptoms of individuals. To avoid deviation of statistical results caused by some individuals undergoing multiple tests in a short period, their duplicate data with the same test result within 1 month was eliminated. Co-detection is defined as a patient having two or more positive nucleic acid tests for respiratory pathogens during the same hospitalization or emergency department visit. The Ethics Committee of the Peking University People’s Hospital approved our study. Moreover, informed consent was waived owing to the retrospective nature of this study. According to the age classification standards established by the National Bureau of Statistics of China, the study population was categorized into two groups: minors (age < 18 years) and adults (age ≥ 18 years).

### Sample collection and testing

The types of specimens included throat swabs and nasal swabs, which were collected by professionals using standardized procedures and transported to clinical laboratories for test. IAV, IBV, RSV, HRV, ADV and MP were detected using a multi-color fluorescent probes real-time PCR Kit (Sansure Biotech Inc. Changsha, China). SARS-CoV-2 were separately detected. Applied Biosystems 7,500 Real-Time PCR System (Thermofisher Scientific Inc., USA) was used for nucleic acid detection of seven respiratory pathogens.

### Statistical analysis

Normality was assessed using the Kolmogorov–Smirnov test. For descriptive statistics, categorical variables were presented as frequencies, while continuous variables were summarized using medians and interquartile ranges (IQR) in this study. To compare the characteristics of different types of respiratory pathogens infections, *Chi*-square tests or Fisher’s exact tests were employed for categorical variables. Continuous variables were analyzed using Kruskal-Wallis tests, with Bonferroni-adjusted pairwise comparisons where overall significance was detected. *p*-value ≤ 0.05 was considered statistically significant. IBM SPSS Statistics 26.0 and R (4.3.2) were utilized for data processing, analysis, and visualization.

## Results

### An increase in respiratory illnesses in Beijing

**Figure 1:**
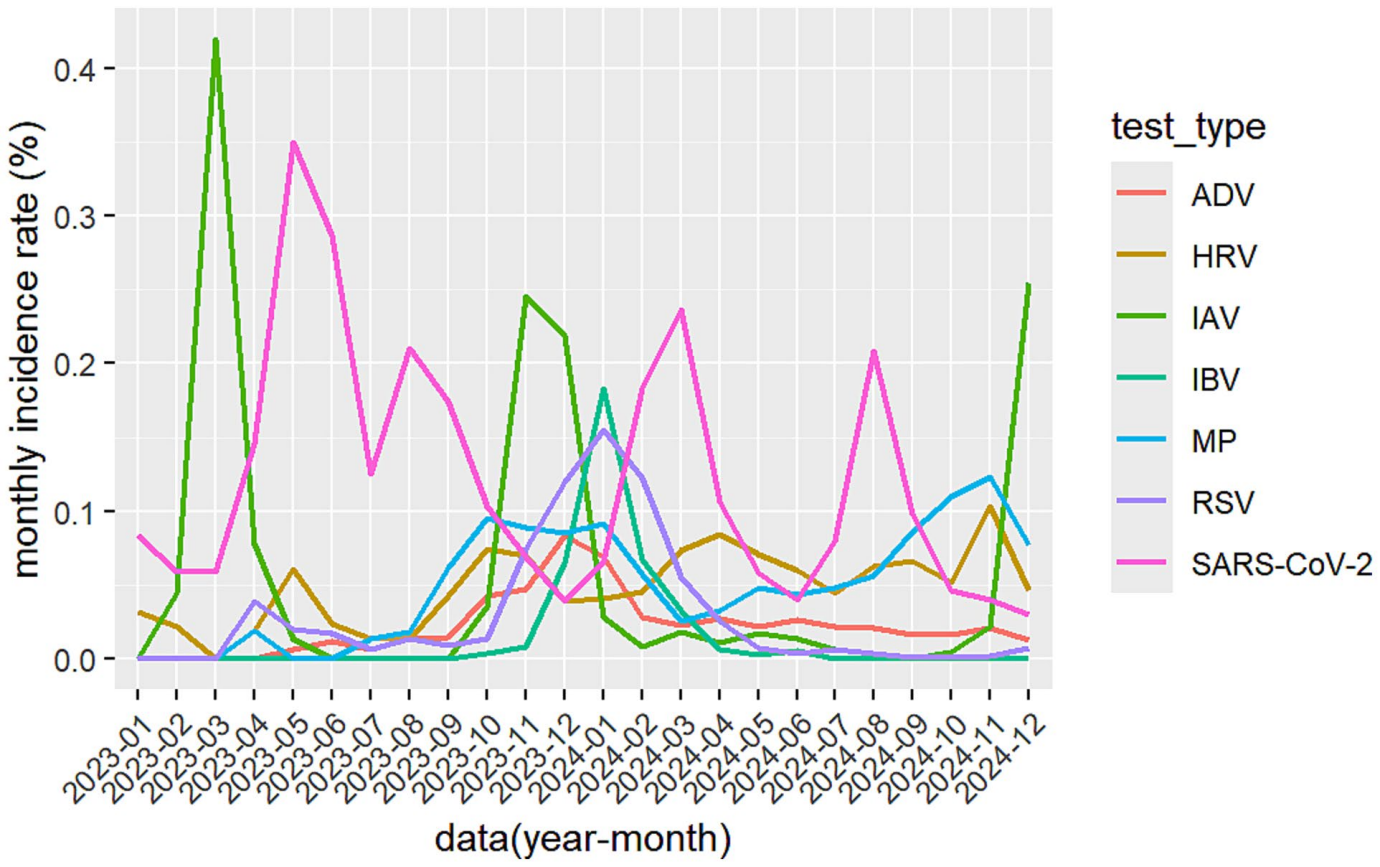
The monthly incidence rate of seven respiratory pathogens. ADV, adenovirus; SARS-CoV-2, severe acute respiratory syndrome coronavirus 2; HRV, human rhinovirus; IAV, influenza A virus; IBV, influenza B virus; MP, mycoplasma pneumoniae; RSV, respiratory syncytial virus.

We calculated monthly respiratory pathogens infection trends from January 2023 to December 2024 as shown in Figure 1, and semi-annual respiratory pathogens positivity rates as shown in Table 1. The volume of the panel of six respiratory pathogen NAT testing showed an increasing trend, reaching a peak of 1,134 cases per month in January 2024. Although it began to decrease in February, it was still close to 600 cases per month, and it reached another testing peak by the winter of 2024 (Supplementary Table 1), these observations suggest that following the pandemic, the detection demand for the panel of six respiratory pathogens has also surged rapidly.

**Table 1:** Semi-annual test positive rate (%) of multiple respiratory pathogens.

TPR%	SARS-CoV-2	ADV	HRV	IAV	IBV	MP	RSV
January-June, 2023	18.0 (937/5197)	0.8 (5/620)	2.7 (17/620)	10.3 (64/620)	0 (0/620)	0.3 (2/620)	1.6 (10/620)
July-December, 2023	11.9 (515/4329)	5.3 (121/2286)	4.6 (106/2286)	14.7 (337/2286)	2.9 (67/2286)	7.4 (170/2286)	6.9 (160/2286)
(χ^2^) *p* value	68.76 (<0.001)	23.67 (<0.001)	4.32 (0.038)	4.20 (0.041)	18.60 (<0.001)	44.32 (<0.001)	25.69 (<0.001)
January-June, 2024	13.2 (670/5083)	3.6 (170/4728)	6.0 (285/4728)	1.7 (81/4728)	6.3 (300/4728)	5.3 (252/4728)	7.3 (348/4728)
July-December, 2024	9.4 (461/4926)	1.8 (85/4738)	6.3 (297/4738)	6.7 (317/4738)	0.02 (1/4738)	8.4 (398/4738)	0.4 (19/4738)
(χ^2^) *p* value	36.91 (<0.001)	29.30 (<0.001)	0.237 (0.626)	145.56 (<0.001)	307.42 (<0.001)	34.88 (<0.001)	307.55 (<0.001)
(total)	13.2 (2,583/19535)	3.1 (381/12372)	5.7 (705/12372)	6.5 (799/12372)	3.0 (368/12372)	6.6 (822/12372)	4.3 (537/12372)

During the two-year study period, SARS-CoV-2 demonstrated distinct epidemic trends, maintaining a positivity rate above 3.0%. Significant infection peaks were observed in May 2023 (35.0%, 424/1213), August 2023 (21.1%, 159/755), March 2024 (23.7%, 289/1220), and August 2024 (20.9%, 241/1155). IAV exhibited seasonal outbreaks predominantly in spring and winter, reaching epidemic peaks in March 2023 (41.9%, 52/124), from November 2023 (24.5%, 119/485) to December 2023 (21.9%, 208/950), and in December 2024 (25.4%, 289/1136). RSV cases began to increase in November 2023, peaking in January 2024 (15.5%, 174/1124), followed by a gradual decline. Throughout the winter season (November to February), RSV positivity rates remained between 7 and 15%. MP maintained positivity rates between 6 and 9% from September 2023 to February 2024, indicating predominant autumn-winter circulation, followed by a decline. However, MP positivity rates increased again starting in September 2024. IBV showed almost no positive cases from early 2023 through October 2023. However, positivity rates increased from November 2023, reaching an epidemic peak in January 2024 (18.3%, 206/1124), followed by a gradual decline, demonstrating winter seasonality. HRV maintained relatively low positivity rates throughout the study period, though overall rates in 2024 were higher than those in 2023. In general, during the autumn and winter of 2023, a large number of patients with fever, cough and sore throat appeared, the infection and co-detection rate of multiple respiratory pathogens has risen sharply.

The testing volume for SARS-CoV-2 remained relatively stable throughout the year without significant fluctuations. The testing volume for the other six respiratory pathogens showed a marked increase starting from the second half of 2023, which persisted through 2024 (Table 1). With the exception of SARS-CoV-2, the detection rates of the other six respiratory pathogens were significantly higher in the second half of 2023 (July–December) compared to the first half (January–June) (*p* < 0.05). However, in 2024, only IAV and MP maintained this trend, while the other respiratory pathogens exhibited opposite epidemic patterns or showed no significant differences, as detailed in Table 1.

### Characteristics of individuals infected by multiple respiratory pathogens

No significant differences were observed in the gender distribution among patients testing positive for the seven respiratory pathogens. Except for IAV, IBV, and MP, which exhibited slightly lower detection rates in males compared to females, all other pathogens showed marginally higher detection rates in males. In the adult group, the proportion of female MP-positive patients was nearly twice that of males, with a male-to-female ratio of 0.52 (146/281). No significant gender differences were observed in other respiratory pathogen-positive patients. The median age range for patients positive with various respiratory pathogens was 23–54 years. Among minors, the median age for most respiratory pathogens was 9 years. Significant differences in both age and gender distribution were observed among patients infected with different respiratory pathogens, with the exception of minors (age < 18), who showed no significant gender disparity (Table 2).

**Table 2:** The median age (IQR) and sex ratio of respiratory pathogens positive individuals.

	Total		Age<18 years		Age≥18 years	
	Median age (IQR)	Sex ratio (M/F)	Median age (IQR)	Sex ratio (M/F)	Median age (IQR)	Sex ratio (M/F)
SARS-CoV-2	54 (34, 69)	1.05 (1, 321/1262)	13 (9, 16)	1.42 (118/83)	57 (37.25, 70)	1.02 (1,203/1179)
ADV	24 (9, 43)	1.17 (205/175)	9 (6, 11)	1.42 (104/73)	42 (35, 59)	0.99 (101/102)
HRV	34 (13, 56)	1.14 (376/329)	9 (5, 13)	1.47 (137/93)	46 (33, 63)	1.01 (139/136)
IAV	49 (29, 67)	0.86 (369/430)	9 (6, 12.75)	1.19 (75/63)	56 (39, 69)	0.80 (294/367)
IBV	35 (14, 47)	0.79 (162/206)	7 (5, 10)	1.08 (52/48)	39 (33, 54)	0.70 (110/158)
MP	23 (10, 41)	0.74 (349/473)	10 (7, 13)	1.06 (203/192)	41 (35, 59.5)	0.52 (146/281)
RSV	40 (17, 63)	1.15 (287/249)	7 (3, 10.25)	1.04 (71/68)	56 (36, 68)	1.19 (216/181)
*P* value	<0.001	<0.001	<0.001	0.322	<0.001	<0.001

**Figure 2:**
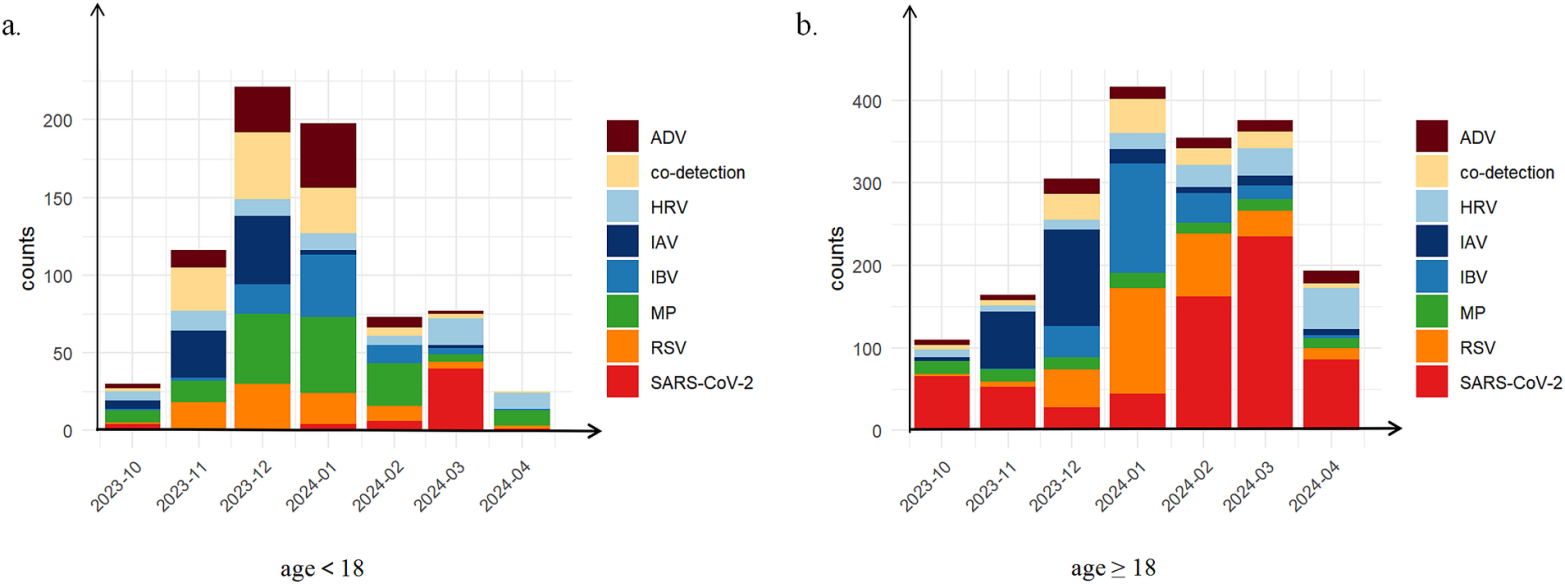
The distribution of positive patient counts across different types of respiratory pathogen infections stratified by age group during the co-circulation period from October, 2023 to April, 2024. **(a)** age<18 years (*N* = 740); **(b)** age≥18 years (*N* = 1920). Eight distinct infection types were investigated: pure infections of severe acute respiratory syndrome coronavirus 2 (SARS-CoV-2), adenovirus (ADV), human rhinovirus (HRV), influenza A virus (IAV), influenza B virus (IBV), mycoplasma pneumoniae (MP), respiratory syncytial virus (RSV); co-detection.

Prior to October 2023, respiratory infections demonstrated relatively homogeneous patterns, predominantly characterized by SARS-CoV-2 cases. From October 2023 to April 2024, a phenomenon of co-circulation and alternating epidemics of multiple respiratory pathogens emerged. During this period, a total of 740 minors and 1920 adults tested positive for respiratory pathogens. Among minor patients, the top three detected pathogens were MP (21.4%, 158/740), co-detection (15.0%, 111/740), and ADV (12.7%, 94/740). In adult patients, the most frequently detected pathogens were SARS-CoV-2 (35.1%, 674/1920), RSV (15.8%, 304/1920), and IAV (12.3%, 236/1920) (Supplementary Table 2). Notably, co-detection ranked as the second most common pattern among minors, indicating a characteristic co-detection epidemic during this period. Although the number of positive cases in adults (1920 cases) significantly exceeded that in minors (740 cases), the number of co-detections showed less disparity (adults: 132 cases; minors: 111 cases). These findings suggest that infection patterns were more diverse in minors compared to adults, as illustrated in Figure 2.

**Figure 3:**
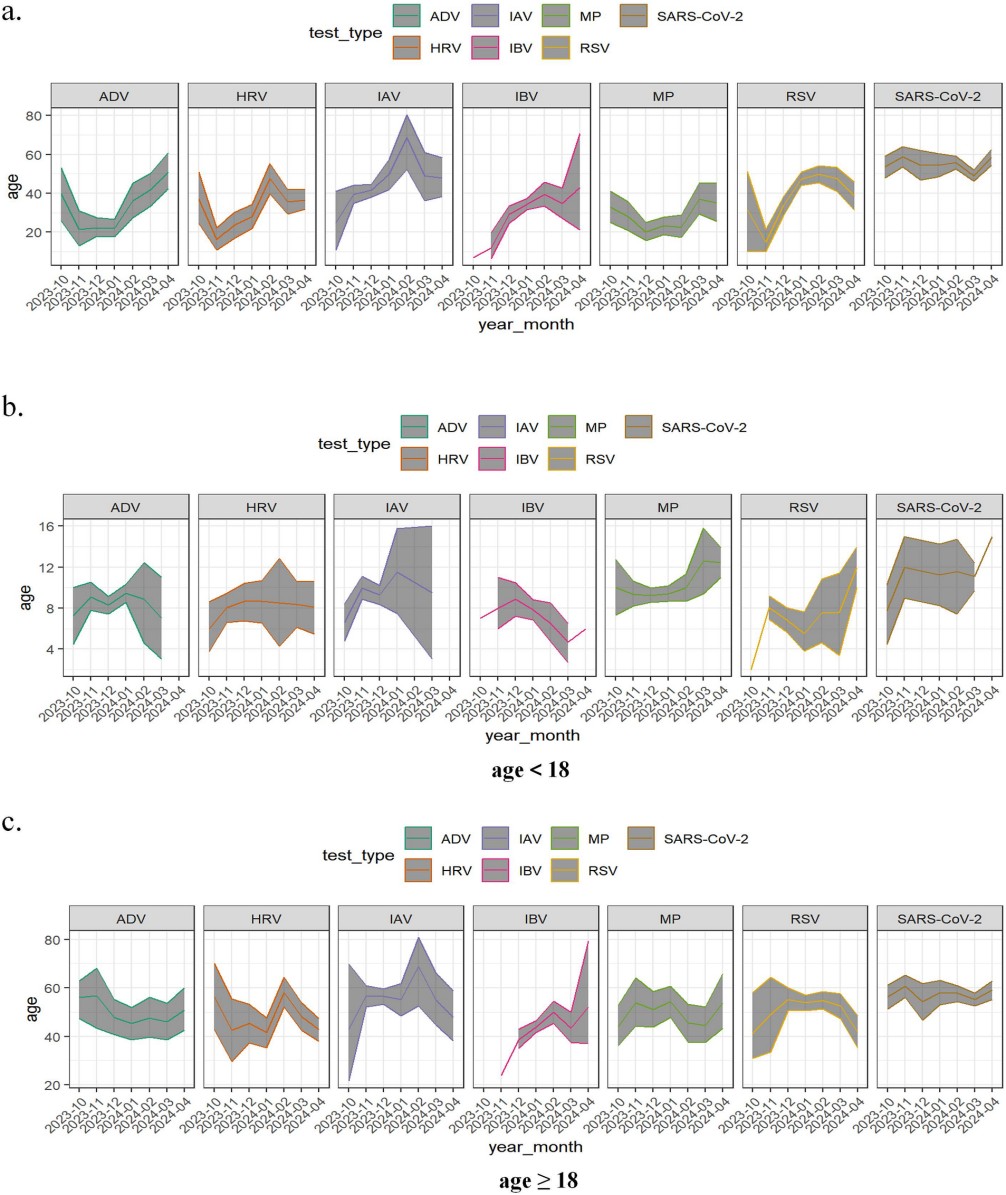
Age changing trace of positive patients stratified by age group during the co-circulation period from October 2023 to April 2024. **(a)** Totally; **(b)** age<18 years; **(c)** age≥18 years. Seven respiratory pathogens were investigated: respiratory syndrome coronavirus 2 (SARS-CoV-2); adenovirus (ADV); human rhinovirus (HRV); influenza A virus (IAV); influenza B virus (IBV); mycoplasma pneumoniae (MP); respiratory syncytial virus (RSV). The line represents the variation in the average age of positive cases across different months. Confidence bands were shaded gray.

Further analysis of the monthly age distribution trends among positive cases from the co-circulation period revealed distinct variations in the predominant age groups affected by different respiratory pathogens across months. While SARS-CoV-2 exhibited minimal fluctuations in its age distribution curve, other pathogens demonstrated notable variations in the age ranges of infected individuals, as shown in Figure 3a. Notably, February 2024 marked a peak in the age distribution of IAV and HRV infections. To better characterize age-related patterns, patients were stratified into minor and adult groups for subgroup analysis. Among minors, SARS-CoV-2 main affected individuals above 10 years, whereas ADV, HRV, IBV, and RSV predominantly infected children below 10 years. In the adult group, the median age of SARS-CoV-2-positive patients was approximately 60 years, while ADV, HRV, IBV, MP, and RSV cases were primarily distributed among individuals below 60 years. Additionally, IAV showed substantial monthly variations in the age distribution of positive cases, as demonstrated in Figures 3b,c.

### Characteristics of co-detection of multiple respiratory pathogens

From January 2023 to December 2024, a total of 324 patients tested positive for two or more respiratory pathogens simultaneously, comprising 167 males and 157 females, with an age range of 0 to 101 years. Among these cases, 17 patients exhibited triple infections, with 13 cases involving RSV co-detected with two other pathogens. Notably, in December 2023, a 7-year-old child tested positive for four respiratory pathogens simultaneously: RSV, HRV, ADV, and MP (as shown in Supplementary Table 3).

**Figure 4:**
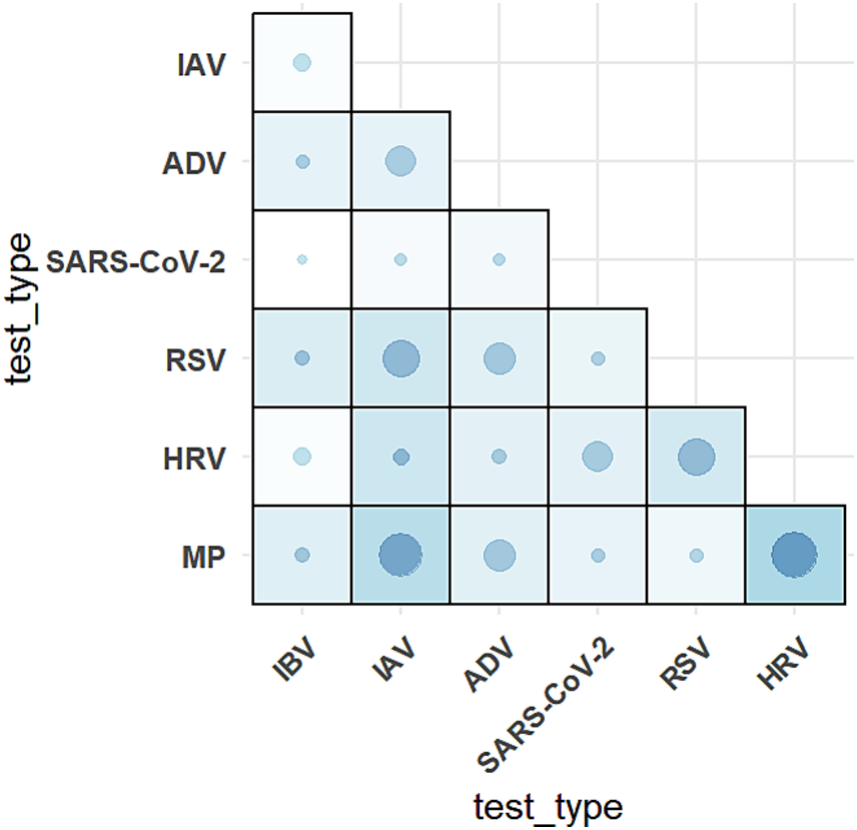
Prevalence of seven respiratory pathogens in co-detection from January, 2023 to December, 2024. Heatmap of respiratory pathogens co-detection. The grid color and the dot color and size represent the number of co-detected patients. Darker grids as well as circles with bigger sizes and darker shades denote a higher number between the pair of pathogens. These seven respiratory pathogens include: respiratory syndrome coronavirus 2 (SARS-CoV-2); adenovirus (ADV); human rhinovirus (HRV); influenza A virus (IAV); influenza B virus (IBV); mycoplasma pneumoniae (MP); respiratory syncytial virus (RSV).

**Figure 5:**
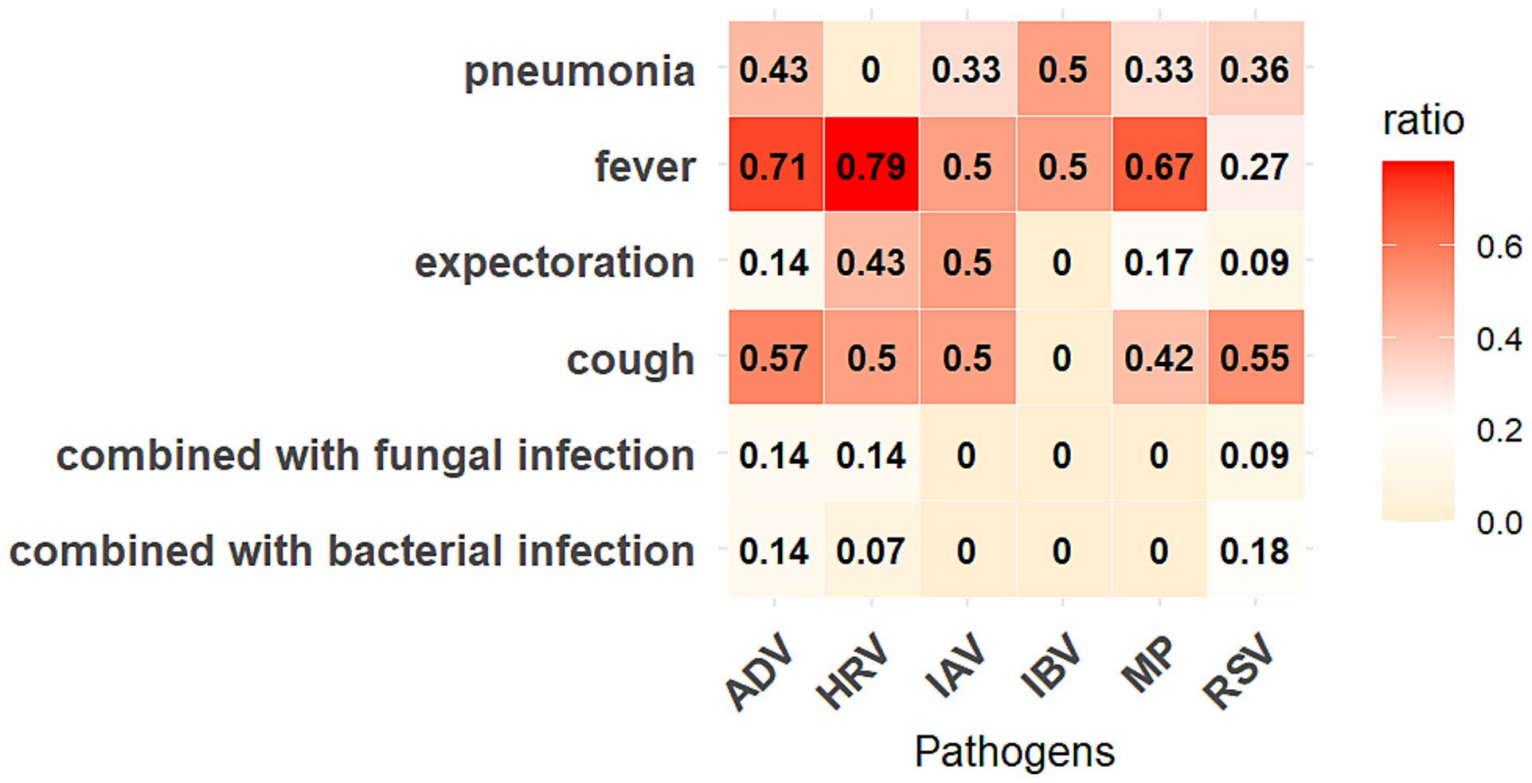
Symptoms of COVID-19 patients co-detected with six other respiratory pathogens. The “ratio” represents the proportion of patients with corresponding symptoms in that co-detected combination.

MP demonstrated the most significant co-detection patterns (Figure 4), with 130 cases (including: MP + HRV (36 cases); MP + RSV (9 cases); MP + SARS-COV-2 (12 cases); MP + ADV (15 cases); MP + IAV (31 cases); MP + IBV (16 cases); MP + HRV + ADV (1 case); MP + HRV + RSV (1 case); MP + IAV + RSV (3 cases); MP + IAV + ADV (1 case); MP + IAV + HRV (1 case); MP + RSV + IBV (2 cases); One quadruple infection). HRV and IAV followed with 116 and 111 co-detection cases, respectively. SARS-CoV-2 showed the fewest co-detection instances (52 cases), all of which were dual infections, primarily occurring in older populations with a median age above 45 years (Supplementary Table 4). Among pediatric patients, the age distribution did not differ significantly across different types of co-detections (Table 3). We further analyzed the clinical symptoms of patients with SARS-CoV-2 co-detection involving six other respiratory pathogens, revealing that fever emerged as the most prevalent clinical manifestation, with over 60% of ADV-, HRV-, and MP-co-detected patients exhibiting this symptom. Notably, pneumonia manifestations were observed in all co-detection combinations except those involving HRV. Furthermore, concurrent bacterial or fungal co-infections were identified in cases of COVID-19 patients co-detected with ADV, HRV, or RSV (Figure 5). Throughout the two-year period, co-detection patterns were observed for all seven respiratory pathogens. Further analysis of dual infection patterns revealed distinct age-related characteristics. ADV, MP, and HRV each exhibited three co-detection combinations with a median age <18 years, indicating a younger age profile for these pathogen combinations. Among minors, the age distribution of co-detected individuals clustered around 10 years. In contrast, the adult group showed a broader median age range of 28–69 years, with a predominance of individuals above 50 years.

**Table 3:** The number and median age (IQR) of individuals co-detected by different respiratory pathogens pairs.

	Total		Age<18 years		Age≥18 years	
	*N*	Median age (IQR)	*N*	Median age (IQR)	*N*	Median age (IQR)
ADV & HRV	14	14 (7.5, 32.5)	7	7 (5.0, 9.0)	7	28 (26.0, 38.5)
ADV & IAV	13	9 (9.0, 65.0)	9	9 (8.0, 9.0)	4	67 (65.8, 69.3)
ADV & IBV	13	39 (35.3, 43.8)	3	6 (5.5, 9.5)	10	41.5 (39, 45.5)
ADV & MP	15	9 (7.3, 14.5)	11	9 (7, 9.5)	4	46.5 (41.3, 54.3)
ADV & RSV	15	19.5 (6.3, 50.8)	7	6 (4.8, 8.3)	8	51 (38.0, 59.0)
SARS-CoV-2 & ADV	7	46 (26, 57.8)	2	10.5 (8.8, 12.3)	5	57 (35.0, 58.0)
SARS-CoV-2 & HRV	14	56 (19.0, 74.0)	2	10 (6, 14)	12	65 (55.0, 76.3)
SARS-CoV-2 & IAV	6	53.5 (32.5, 68.5)	1	15 (15.0, 15.0)	5	69 (40.0, 70.0)
SARS-CoV-2 & IBV	2	49.5 (42.8, 56.3)	0	0 (0.0, 0.0)	2	49.5 (42.8, 56.3)
SARS-CoV-2 & MP	12	66 (52.0, 78.0)	2	13.5 (13.3, 13.7)	10	65 (52.5, 75.5)
SARS-CoV-2 & RSV	11	62 (52.5, 71.5)	0	0 (0.0, 0.0)	11	62 (52.5, 71.5)
HRV & IAV	23	26 (13.0, 39.3)	8	11.5 (5.0, 14.0)	15	52 (41.5, 60.0)
HRV& IBV	4	31 (6.8, 56.3)	2	5.5 (4.3, 6.8)	2	58.5 (56.3, 60.8)
HRV & MP	36	10 (8.0, 20.0)	28	9 (5.0, 10.25)	8	41.5 (31.3, 45.0)
HRV& RSV	21	12 (10.0, 37.0)	14	10 (5.8, 11.8)	7	50 (37.0, 64.0)
IAV & IBV	4	32 (31.5, 35.5)	0	0(0.0, 0.0)	4	34 (31.8, 36.8)
IAV & MP	31	16.5 (8.0, 69.0)	9	8 (6.0, 13.0)	22	50 (35.3, 73.5)
IAV & RSV	22	53.0 (11.3, 64.8)	7	7 (4.0, 9.5)	15	62 (53.0, 72.0)
MP & IBV	16	9.5 (8.0, 12.3)	14	9 (8.0, 11.0)	2	37 (32.5, 41.5)
RSV & IBV	18	40.0 (36.0, 64.0)	2	6 (3.5, 8.5)	16	48.5 (36.0, 64.5)
RSV & MP	9	51 (7.0, 62.0)	4	6 (4.8, 7.5)	5	62 (61.0, 85.0)
*p* value		<0.001		0.370		0.006

Although the number of adult positive cases significantly exceeded that of minors (4,857 vs. 1,338), minors outnumbered adults in several specific co-detection combinations: ADV + IAV (9 vs. 4), ADV + MP (11 vs. 4), HRV + MP (28 vs. 8), HRV + RSV (14 vs. 7), and MP + IBV (4 vs. 2). Detailed data are presented in Table 3.

## Discussion

This study characterized the post-pandemic prevalence dynamics of seven respiratory pathogens in Beijing area, documenting their transition to sustained co-circulation from autumn 2023 through July 2024. We particularly investigated two aspects: the demographic patterns in single-pathogen infections and epidemiological features of multi-pathogen co-detection during this transitional phase.

The implementation of NPIs during 2020–2022 significantly suppressed influenza transmission globally, as reported in multinational surveillance studies (4, 12, 13). This is because most respiratory pathogens are mainly transmitted by droplets, hands and aerosols, which are similar to SARS-CoV-2 (14). From the beginning of 2023, the impact of the post-pandemic has attracted people’s considerable attention. The post-NPI resurgence of respiratory infections since 2023 has emerged as a critical public health concern. While international studies have mapped post-pandemic transmission shifts (15, 16), there are still a limited number of relative reports in China due to its distinctive epidemiological landscape – characterized by delayed reopening (December 2022) and pronounced north–south climatic disparities (10, 17, 18). Our investigation addresses this knowledge gap through three key contributions: First, it establishes baseline transmission patterns for respiratory pathogen resurgence in northern China’s megacity setting. Second, it reveals age-stratified susceptibility profiles across pathogen types. Third, it provides the systematic analysis of co-detection demographics in China’s post-pandemic era.

Our surveillance revealed distinct epidemiological patterns among respiratory pathogens during 2023–2024. IAV demonstrated an unprecedented resurgence in early 2023, reaching a peak positivity rate exceeding 40% – a level consistent with Beijing’s record-breaking dual epidemic waves reported that year (10). Following the initial spring 2023 surge, RSV exhibited delayed transmission dynamics, with explosive spread occurring in January 2024 rather than displaying the off-season epidemics reported post-NPI relaxation in other regions (19–21). While RSV typically affects susceptible populations such as young children and the older adults, our findings highlight a distinct epidemiological pattern of RSV infections, with adults emerging as the predominant affected demographic. This trend aligns with Brazilian surveillance data that documented comparable RSV prevalence across all age groups during the COVID-19 pandemic (22). Several studies have demonstrated that spatial mobility of populations influences the transmission dynamics of respiratory pathogens (23, 24). COVID-19 resurgence events may be associated with sociodemographic factors: the August–September 2023 surge coincided with mass student mobility during summer vacation and academic reopening, while the February 2024 peak (predominantly affecting adults) correlated with China’s Spring Festival migrations.

Respiratory pathogen infections predominantly affect pediatric populations. However, our analysis revealed distinct age-related epidemiological patterns during the co-circulation phase (2023–2024). While MP and ADV exhibited median infection ages below 18 years, other pathogens primarily infected adults, this contradicted established epidemiological profiles (25). This divergence may be caused by post-COVID-19 immunological alterations, including prolonged immune dysregulation in convalescent individuals (2), or immunity debt resulting from extended NPI implementation (26). Furthermore, institutional sampling bias likely contributed to this observation, as our tertiary general hospital predominantly serves adults, whereas pediatric cases typically present at specialized children’s hospitals (11, 27). Notably, 2023 surveillance data revealed high rates of macrolide-resistant MP strains among pediatric patients, consistent with emerging antimicrobial resistance trends (28). To clarify age-specific susceptibility, we stratified populations into minors (<18 years) and adults during co-circulation period. Although minors demonstrated higher overall pathogen positivity rates (Figure 2), confirming their enduring vulnerability, our stratification aligned with recent reports of upward age-shifting in susceptibility peaks, particularly among older children (20, 25, 27). These findings underscore the complex interplay between pandemic-related behavioral changes, pathogen evolution, and healthcare-seeking patterns in reshaping post-COVID-19 respiratory disease epidemiology.

Beijing entered a sustained phase of respiratory pathogen co-circulation in October 2023, characterized by concurrent positivity for seven pathogens over multiple months. Pediatric populations exhibited particularly diverse infection profiles during this period. While global studies reported co-infection rates of 5–13% for SARS-CoV-2 with other pathogens—predominantly involving IAV, HRV, ADV and IBV, and children are more likely to be coinfected than adults, resulting in more severe symptoms like breathing difficulties and even increasing the mortality (29–31). It’s reported that Germany and United States experienced a co-circulation of SARS-CoV-2, influenza, and RSV during the winter of 2022/2023 (32, 33). A combination of SARS-CoV-2 and IAV co-infection associated with exacerbated disease severity, increased mechanical ventilation requirements, and higher ICU admission rates across multiple studies (10, 34, 35). In our study, COVID-19 patients co-detected with six other respiratory pathogens predominantly presented with fever and cough, with pneumonia developing in over 30% of cases across five co-detection groups. Notably, MP emerged as the primary co-detection driver in our cohort (January 2023–December 2024), followed by HRV and IAV. Despite lower testing volumes among minors, respiratory pathogens co-detection prevalence paralleled adult rates. A striking case involved a 7-year-old child with quadruple pathogen detection within 1 month. These observations align with Beijing-specific research documenting elevated IAV/RSV co-detection frequencies and amplified symptom severity in SARS-CoV-2 co-infections during 2023 autumn-winter peaks (10).

There are some limitations in this study. First of all, this was a single-center retrospective study, the sample data were exclusively obtained from patients at Peking University People’s Hospital, which may not fully represent the overall respiratory pathogen epidemiology in Beijing. However, the findings can be considered alongside other regional studies for comprehensive reference. Secondly, this investigation was limited to seven common respiratory pathogens, which may not fully capture the complete spectrum of acute respiratory infection (ARI) pathogens. Additionally, the study did not include analysis of pathogen genotypes. In addition, this research did not incorporate hospitalization rate and ICU admission. Inclusion of such data could have provided valuable insights into the severity and characteristics of clinical manifestations associated with different respiratory pathogen epidemics.

## Conclusion

Long-term NPI removal may lead to a rebound of the epidemic. We observed the prevalence of respiratory viruses in Beijing for more than 1 year after the end of the epidemic, and found that from the autumn and winter of 2023 to the spring of 2024, the detection burden of the panel of six respiratory pathogens increased significantly, multiple respiratory viruses cocirculated, and the infection forms of the minor group more diversified. MP and HRV are susceptible to be co-detected with other respiratory pathogens. Nevertheless, we believe that this report provides clinical doctor with scientific evidence for respiratory pathogens infections in Beijing area.

## Data Availability

The raw data supporting the conclusions of this article will be made available by the authors, without undue reservation.
